# Drug Development Targeting the Ubiquitin–Proteasome System (UPS) for the Treatment of Human Cancers

**DOI:** 10.3390/cancers12040902

**Published:** 2020-04-07

**Authors:** Xiaonan Zhang, Stig Linder, Martina Bazzaro

**Affiliations:** 1 Masonic Cancer Center and Department of Obstetrics, Gynecology and Women’s Health, University of Minnesota, Minneapolis, MN 55455, USA; xiaonan.zhang@ki.se; 2 Department of Oncology-Pathology, Karolinska Institutet, 171 77 Stockholm, Sweden; stig.linder@liu.se; 3 Department of Immunology, Genetics, and Pathology, Uppsala University, 751 05 Uppsala, Sweden; 4 Department of Medical and Health Sciences, Linköping University, SE-58183 Linköping, Sweden

**Keywords:** ubiquitin, cancer, targeted therapy, chemoresistance

## Abstract

Cancer cells are characterized by a higher rate of protein turnover and greater demand for protein homeostasis compared to normal cells. In this scenario, the ubiquitin–proteasome system (UPS), which is responsible for the degradation of over 80% of cellular proteins within mammalian cells, becomes vital to cancer cells, making the UPS a critical target for the discovery of novel cancer therapeutics. This review systematically categorizes all current reported small molecule inhibitors of the various essential components of the UPS, including ubiquitin-activating enzymes (E1s), ubiquitin-conjugating enzymes (E2s), ubiquitin ligases (E3s), the 20S proteasome catalytic core particle (20S CP) and the 19S proteasome regulatory particles (19S RP), as well as their mechanism/s of action and limitations. We also discuss the immunoproteasome which is considered as a prospective therapeutic target of the next generation of proteasome inhibitors in cancer therapies.

## 1. The Ubiquitin–Proteasome System Is Essential for the Maintenance of Protein Homeostasis

In mammalian cells, protein turnover must be strictly regulated as nearly one-third of the newly synthesized proteins are rapidly degraded with a half-life no more than 10 min [1]. At the same time, proteins that are damaged or misfolded also require prompt degradation to keep a functional cellular metabolism [2]. The ubiquitin–proteasome system (UPS) is a specialized proteolysis system that controls protein degradation and plays an essential role in cellular protein homeostasis [3,4]. Evidence has revealed that up to 80% of cellular proteins are degraded through the UPS which speaks about its importance not only in the regulation of protein homeostasis, but also in the management of numerous cellular regulators relating to DNA damage and repair, cell proliferation and survival, cell differentiation as well as drug resistance [5,6,7,8,9,10,11].

A series of essential components—ubiquitin, ubiquitin-activating enzymes (E1s), ubiquitin-conjugating enzymes (E2s), ubiquitin ligases (E3s), deubiquitinating enzymes (DUBs), as well as the 26S proteasome—constitute the UPS [12,13]. Ubiquitin is a highly conserved 76 amino acid proteins that oversees marking to-be-degraded proteins by covalent attachment through an isopeptide bond between the carboxy glycine residue (G76) of ubiquitin to the ε-amino groups of lysine residues [14]. The 26S proteasome is a large multi-subunit shredder where ubiquitin-tagged proteins are degraded into smaller peptides which are either further degraded into amino acids or recycled for further application during other cellular metabolic processes. For example, Cyclin B1 is degraded by proteasome into multiple short chains to regulate cell cycle [15,16]. Oxidized histone protein Htb2, a core component of the nucleosome, which is critical for transcription and cell cycle, is recognized and linked by Lysine Residue 48 (K48) and further degraded by the proteasome [17,18]; DbpB (also named Y-box protein 1), a transcription factor, is reported to selectively recognize the Y-box promoter element. Studies showed that its terminal 105-amino-acid-long fragment is removed after a specific proteolytic cleavage by the proteasome complex [19,20]; NF-κB (nuclear factor kappa-light-chain-enhancer of activated B cells) is located outside the nucleus and is reported to be involved in DNA transcription as well as cell survival [21,22]. The NF-κB p105 is the precursor of NF-κB p50. It is evident that NF-κB p105 is cleaved and selectively degraded at the C-terminus by proteasome, generating the active form of NF-κB p50 [23]. Products of UPS degradation can also be further degraded into single amino acids by aminopeptidases [24]. Aminopeptidases are the class of enzymes that catalyze the final steps in the ubiquitin–proteasome pathway by breaking down shorter peptides (<5 residues) into even smaller fragments [25]. Many, but not all, of aminopeptidases, are zinc metalloenzymes, such as leucine aminopeptidases (lAPs) and methionine aminopeptidases (metAPs) [26,27]. Studies showed that blocking the activity of the aminopeptidases by inhibitor of bestatin could generate a major accumulation of peptides which are ∼2–5 residues long [28].

**Figure 1:**
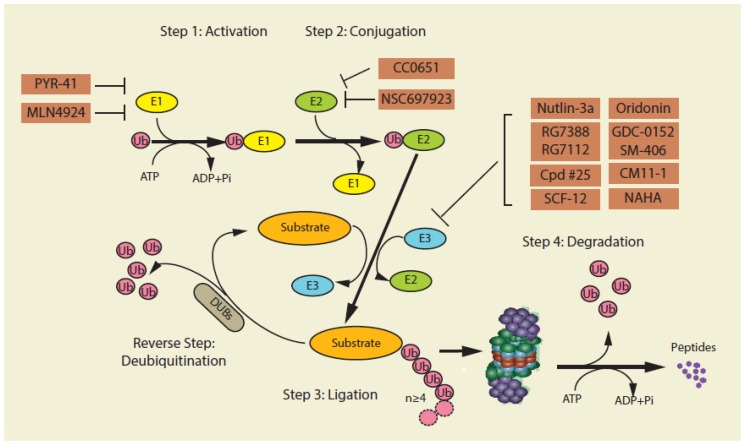
An overview of the ubiquitin–proteasome system (UPS). UPS-mediated protein degradation requires a series of essential components: ubiquitin, ubiquitin-activating enzymes (E1s), ubiquitin-conjugating enzymes (E2s), ubiquitin ligases (E3s) and the 26S proteasome. Within the UPS a reversed reaction of protein deubiquitylation catalyzed by deubiquitinases (DUBs) is also performed. Proteasome inhibitors targeting different components of the UPS are included (additional inhibitors targeting the 26S proteasome and the immunoproteasome are shown in Figure 3 and Figure 4, respectively).

The 26S proteasome contains one/two 19S regulatory particles (19S RP) which mainly regulate the translocation of ubiquitinated proteins to the 20S CP and one 20S core particle (20S CP) in which proteolysis finally occurs [29,30]. In general, two main processes are associated with the process of degradation of proteins by the UPS: (1) tagging the to-be-degraded proteins by polyubiquitination (normally more than four ubiquitins), and (2) proteolytic degradation of the polyubiquitinated protein by the 26S proteasome complex [31,32]. Each step incorporates an intricate and complex spectrum of protein interactions and biochemical reactions (Figure 1).

### 1.1. Tagging the to-Be-Degraded Proteins by Polyubiquitination

This step, typically considered as a post-translational modification of lysine residues, involves the UPS components of ubiquitin, ubiquitin-activating enzymes (E1s), ubiquitin-conjugating enzymes (E2s), ubiquitin ligases (E3s), and deubiquitinating enzymes (DUBs). The human genome contains two E1 genes which are mainly responsible for ubiquitination—UBA1 (UBE1) and UBA6 (UBE6). UBA1 (UBE1) and UBA6 (UBE6) are expressed ubiquitously and have been thought to be interchangeable in many ubiquitination events by transferring Ub to a shared pool of E2s and E3s [33,34]. There are about fifty E2 enzymes and more than six hundred E3 enzymes, each of which has a specific function of modulating the activity of downstream protein substrates [12,13]. Firstly, the 76-amino acid ubiquitin polypeptide is activated through the assistance of the activating enzyme E1. Activation occurs following a covalent linkage between the carboxyl-terminus of ubiquitin and a cysteine residue convey on E1 to form a thioester bond (E1-Ub). Secondly, the ubiquitin activated by E1 is designed to be presented to an E2 ubiquitin-conjugating enzyme (E2-Ub). Lastly, a substrate-specific E3 ligase enzyme transfers the ubiquitin from E2 to a specific substrate protein. Since E3 proteins are responsible for recognizing and binding to a specific substrate, it is not surprising that over six hundred E3 enzymes appear to be encoded by the human genome. These E3 ligases are generally classified into three groups of the ‘really interesting new gene’ (RING) class, the ‘homologous to E6-AP carboxy-terminus’ (HECT) class, and the ‘RING-between-RING’ (RBR) class. The RING class, the greater part of the E3 ligases, acts as a mediator by proving a docking site to bring the to-be-degraded substrates close to E2-Ub, thus allowing E2-Ub to transfer Ub directly to the substrates without forming thioester bonds with ubiquitin [35]. The HECT class undergo a catalytic cysteine-dependent transthiolation reaction with E2–Ub, forming a covalent E3–Ub intermediate [36,37]. RBR class have a canonical RING domain linking to an accessorial domain [38]. The step of tagging the to-be-degraded proteins normally needs to proceed at least four cycles in order to form substrate-polyubiquitins which could be recognized by the 26S proteasome complex [14,31,33,39].

It is important to point out that ubiquitin contains seven different lysine residues (Lys6, Lys11, Lys27, Lys29, Lys33, Lys48 and Lys63), any of which can be covalently linked by other ubiquitin molecules and determine a specific fate of the substrate protein. In general, Lys-48 and Lys-11 attached chains are further involved in proteasome degradation [40,41], whereas those linked by Lys63-linked chains generally undergo nonproteolytic processes such as DNA repair, DNA replication and signal transduction [42]. Other linkage types are less well understood so far, even though reports have shown that polyubiquitinated chains covalently linked by Lys6, Lys27, Lys29, or Lys33 are reported to target proteins for proteasome-mediated degradation [43]. The process of ubiquitination is highly dynamic and can be reversed by enzymes known as deubiquitinases (DUBs). Until now, more than ninety deubiquitinases (DUB) have been discovered which are generally classified into five different groups based on the presence of conserved catalytic domains: the ubiquitin-specific proteases (USP), ubiquitin-C terminal hydrolases (UCH), Machado–Joseph domain (MJD), ovarian tumor domain (OTU), and the Jab1/MPN (JAMM) class [44,45]. It is worth noting that DUBs are increasingly shown to play essential roles in the initiation and progression of multiple cancer types [46].

### 1.2. Proteolytic Degradation of the Polyubiquitinated Protein by the Proteasome Complex

Proteins that have been properly polyubiquitinated from the first step are further recognized by the 19S RP where the poly-Ub groups are removed from the substrates [47]. As the proteasomal channel is too narrow for a folded protein to go through to 20S CP, it is assumed that the 19S particle also unfolds substrates and helps to insert them into the 20S CP for further proteolysis. The energy required in steps of the opening channel and unfolding substrates are supplied by six different ATPase subunits in the base of the 19S RP [31]. The 20S CP includes four layers of ring-like structures [40]. The outer ring layers are composed of seven “alpha” subunits, α1-α7 and the inner “beta” rings contain seven ‘beta’ subunits, β1-β7. The β1 subunits present caspase-like (C-L) proteolytic activity, the β2 subunits have trypsin-like (T-L) activity and the β5 subunits exhibit chymotrypsin-like (CT-L) activity. Following substrate degradation in 20S CPs, short peptides generated from the degraded substrates are recycled and reused for other cellular functions [48].

## 2. The UPS Affects Tumorigenesis, Tumor Metabolism and Survival

Several evidences indicate that cancer cells are highly dependent on a functional UPS system for tumor initiation, tumor metabolism and survival. Thus, components of the UPS have attracted extreme attention for the treatment of cancer in the last decades [49,50].

### 2.1. The UPS and Tumorigenesis

As expression levels of proteins regulating the cell cycle are often under the control of the UPS, aberrancies in the UPS pathway can result in abnormal cell-cycle control and contribute to tumor initiation and development. Kip1, for instance, is an inhibitor of the cyclin-dependent kinase (Cdk) whose levels are “high” in quiescent cells. During tumorigenesis, Kip1 levels drop due to its proteasome-mediated degradation [51,52]. High expression levels of mutant p53 have been reported in human cancers but not in non-transformed cells. These high expression levels are accompanied by deregulation of E3 ubiquitin ligases Murine Double Minute 2 (MDM2) [53,54], suggesting a role of the UPS in the regulation of protein levels of mutant p53 in cancer cells. F-box/WD repeat-containing protein 7 (FBXW7), a general tumor suppressor in human tumorigenesis, is another key E3 ubiquitin ligase. Mutations on this ligase cause an accumulation of MYC and upregulation of m-TOR at the early stage of transformation [35,55,56]. In order to escape proteotoxic stress which normally accompanies fast protein turnover and high proliferative rate, 26S proteasome assembly is significantly stimulated in the process of tumorigenesis [57]. 

### 2.2. The UPS and Tumor Metabolism

Several studies have suggested that the UPS modulates the physiology and the morphology of mitochondria, the powerhouse of mammalian cells, by ubiquitinating the outer mitochondrial membrane (OMM) proteins, including BAX, DRP1, MFN1/2, and VDAC [58,59,60,61,62]. Another study has shown that the UPS pathway plays an indispensable role in the regulation of mitochondrial energy metabolism by regulating the turnover of several mitochondrial oxidative phosphorylation (OXPHOS) proteins such as the succinate dehydrogenase subunit A (SDHA), the mitochondrial respiratory complex II [63,64]. Findings also indicate a mechanism of crosstalk between the proteasome and autophagy pathway [65,66,67,68,69]. This includes the degradation of synaptosomal-associated protein 29 (SNAP29) and syntaxin 17 (STX17) by the ubiquitin-independent 20S proteasome [70]. 

**Figure 2:**
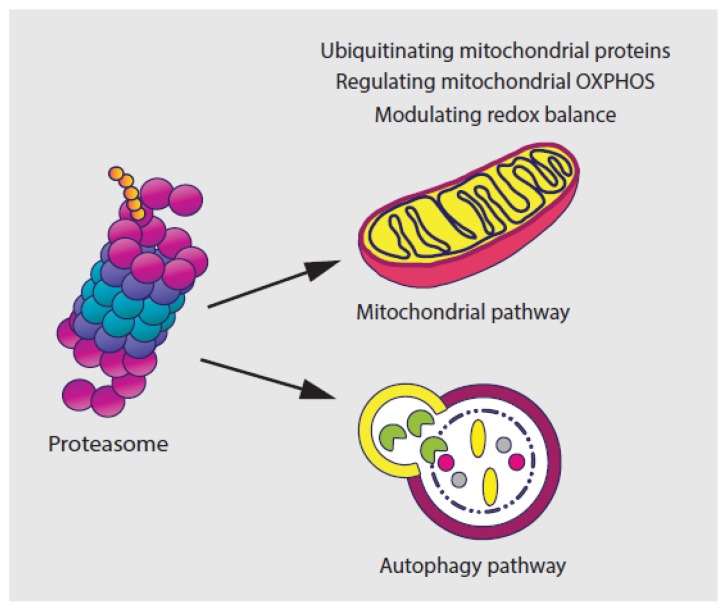
UPS plays a role in regulating tumor metabolism. The UPS, especially the 26S proteasome complex, modulates both mitochondrial morphology and dynamics as well as cross-talks with the autophagy pathway.

The UPS, especially the 26S proteasome complex, is also essential in regulating redox balance by recognizing and removing oxidized, damaged or misfolded proteins [71,72]. The inhibition of proteasome function leads to the induction of oxidative stress, because of excessive production of reactive oxygen species (ROS), which is mainly from mitochondria [71,72,73,74]. On the other hand, continuous exposure to ROS also affects the function of the proteasome [75]. Proteasome-mediated degradation has been shown to be enhanced more than 10-fold upon exposure to H2O2 or O2- [76]. However, acute oxidative stress caused by environmental insults or mitochondrial defects results in the destruction of 26S proteasome activity and rapid disassembly of 26S proteasomes into 20S CP and 19S RP 19S RP subunits [77]. Due to this, the interplay between the proteasome and oxidative stress needs to be strictly balanced for cells to maintain the basic cellular metabolism (Figure 2).

### 2.3. UPS and Tumor Survival

The UPS system could also regulate the fate of cancer cells by modulating the proapoptotic factors of the Bcl-2 superfamily. Mcl-1, an anti-apoptotic protein, is essential for survival and reported to be regulated by TRIM17, MULE and FBW7 of the E3 ligases of the UPS [78,79,80]. Bim, another pro-apoptotic member which regulates stress-induced signals to the core apoptotic machinery [81,82], has been proved to be regulated by the UPS mediated by MAPK/ERK [83,84]. The toxic signaling of TNFα and other death receptors are reported to have multiple sites regulated by the UPS [85].

## 3. Inhibitors of the UPS in Cancer Therapies

As the UPS system is important in regulating aspects of cellular pathways in cancer cells, such as tumor initiation and progression, inhibiting the activities of different components of the UPS has been proposed as a promising therapeutic strategy for the treatment of cancer. Here, we described inhibitors targeting different components of the UPS which are currently at different development stages in clinical studies (Table 1, Table 2 and Table 3).

### 3.1. Inhibitors of Ubiquitin-Activating Enzymes (E1s)

As only two E1s have been reported so far and the step of ubiquitin activation is just the start process of protein degradation, it is important to find inhibitors targeting other enzymes rather than E1s. PYR-41, a pyrazone derivative, is the first cell-permeable inhibitor targeting the E1 enzyme UBA1. This compound is able to irreversibly bind to the active cysteine in UBA1 and abrogate its catalytic activity [86]. The mechanism through which PYR-41 causes cell death is via p53-mediated apoptosis. Thus, its use is particularly promising for the treatment of cancers characterized by p53 mutations [87,88]. Recently, another inhibitor found to be targeting the E1 activation step is MLN4924 (Pevonedistat), a small molecule inhibitor of the E1 NEDD8-activating enzyme [89,90,91,92,93,94]. This small molecule is an adenosine sulfamate analog that covalently binds the nucleotide-binding site of NAE and generates a NEDD8-MLN4924 adduct that further undermines the cullin-RING ligase-mediated protein turnover leading to apoptosis in cancer cells by accumulating proteins of p27, NRF2, CDC25A, HIF1α and IκB [89]. MLN4924 was also reported to inhibit angiogenesis during tumor development [95]. MLN4924 is being currently evaluated for the treatment of patients diagnosed with both hematological and solid tumors [96,97]. Please see Table 1. 

**Table 2:** Inhibitors of the constitutive proteasome complex.

Compounds	Target	Modes of Action	Targeted Cancer Types in Preclinical Studies	Targeted Cancer Types in Clinical Studies or Therapies	Other Disease	Ref.
**Inhibitors targeting 20S core particle of the proteasome**						
**Bortezomib**	β5 > β1	Inhibits the chymotrypsin-like activity of the proteasome by reversible binding to the β5 subunit thus inhibits proteasomal activity and leads to accumulation of polyubiquitinated proteins in cells		Multiple Myeloma Mantle cell lymphoma Acute myeloid leukemia lung cancers hepatocellular carcinoma Intrahepatic Cholangiocarcinoma Relapsed/Refractory Multiple Myeloma Neuroblastoma Colorectal Cancer Head and Neck Cancer Thyroid Carcinoma *More cases to https://clinicaltrials.gov*	Haemolytic anaemia Immune thrombocytopenia Lung disease Cold agglutinin disease Amyloidosis Macroglobulinemia	[98,99,100,101,102,103,104]
**Carfilzomib**	β5	Covalent bonds to proteasome catalytic subunits, predominantly β5		Multiple myeloma Relapsed and/or refractory multiple myeloma Lymphoma Chronic lymphocytic leukemia Thyroid cancer Refractory renal cell carcinoma Lung cancer *More cases to https://clinicaltrials.gov*	Pulmonary arterial hypertension	[105,106,107,108,109,110,111,112]
**Ixazomib**	β5 > β1	First orally bioavailable proteasome inhibitor drug, predominantly targeting β5		Multiple myeloma Refractory or relapsed multiple myeloma Acute myeloid leukemia Relapsed refractory acute myeloid leukemia Hodgkin and T-cell lymphoma Mantle cell lymphoma Non-hematologic malignancies lymphoma Breast cancer Glioblastoma Bladder cancer Renal cell carcinoma Waldenstrom macroglobulinemia Solitary osseous plasmacytoma *More cases to https://clinicaltrials.gov*	Al amyloidosis Autoimmune cytopenia HIV Lupus nephritis Kidney diseases	[113,114,115,116,117,118,119]
**Oprozomib**	β5 > β1	A structural homologue of CFZ, orally available and applied to patients with relapsed after receiving BTZ- and CFZ-based therapies		Multiple Myeloma Relapsed and/or refractory multiple myeloma Hepatocellular carcinoma Waldenstrom macroglobulinemia Non-central nervous system malignancies	No reported applications	[120,121,122,123]
**Marizomib**	β5 > β2 > β1	Irreversibly inhibits the activity of proteasome and more effectively induces apoptosis in tumor cells from MM and chronic lymphocytic leukemia patients, while shows a lower toxicity to normal cells than BTZ		Multiple Myeloma Relapsed and/or refractory multiple myeloma Ependymoma Non-small Cell Lung Cancer Pancreatic Cancer Melanoma Lymphoma Glioblastoma	No reported applications	[124,125,126,127,128]
**Inhibitors targeting 19S regulatory particle of the proteasome**						
**IU1** **IU1-47**	USP14	Targets the thiol group in the active cysteine site in USP14 protease and significantly decrease cell proliferation, migration, and invasion.	Breast cancer Lung cancer		No reported applications	[129,130]
**b-AP15**	USP14 UCHL5	Targets both UCHL5 and USP14, disrupts the aggresome formation in cancer cells by activating caspase to further induce apoptosis relating to an upregulation of oxidative stress	Acute myeloid leukemia Multiple myeloma Large b cell lymphoma Mantle cell lymphoma Neuroblastoma Prostate cancer Breast cancer Lung cancer Head and neck cancer Colon cancer Ovarian cancer		No reported applications	[131,132,133,134,135,136,137,138,139,140,141]
**VLX1570**	USP14 UCHL5	An analog of b-AP15, more effective than b-AP15 in inhibiting tumor progression	See targeted cancer types of b-AP15	Multiple Myeloma	No reported applications	[142,143,144,145]
**RA-9**	USP14	Reacts with the sulfurs in the active site cysteine and inhibits proteasome-associated DUBs	Breast cancer Ovarian cancer Cervical cancer		Rheumatoid arthritis	[146,147]
**WP1130**	UCHL5 USP14 USP9X	Directly inhibits USP9X in addition to UCHL5 and USP14, induces apoptosis and prevents drug resistance in malignancies through Mcl-1 degradation	Acute myeloid leukemia Chronic myelogenous leukemia Human mesothelioma Lung cancer Colon cancer Prostate cancer Hepatocellular carcinoma		No reported applications	[148,149,150,151,152,153,154]
**OPA**	RPN11	A zinc ion chelator, inhibits the activity of RPN11 metal-containing enzymes of 19S and induces apoptosis including cell lines which are BTZ resistant	Multiple myeloma Hepatocellular carcinoma Cervical cancer Breast carcinoma		Sarcoidosis	[155,156,157,158,159,160,161]
**8TQ**	RPN11	A strong RPN11-specific inhibition of proteasome 19S subunit and is a potent apoptosis inducer in MM cells	Lung carcinoma Colon cancer		No reported applications	[162]
**Thiolutin**	RPN11	The reduced form of Thiolutin is an inhibitor of JAB1/MPN/Mov34 (JAMM) domain-containing metalloprotease RPN11 by chelating Zn^2+^-ions which is specifically toxic to cancer cells by hampering protein turnover	Only in cell free system		No reported applications	[163]

**Table 3:** Inhibitors of immunoproteasome complex.

Compounds	Target	Modes of Action	Targeted Cancer Types in Preclinical Studies	Targeted Cancer Types in Clinical Studies or Therapies	Other Disease	Ref.
**ONX-0914**	β5i	The first epoxyketone-based peptidyl immunoproteasome selective inhibitor towards β5i			Rheumatoid arthritis (mouse model)	[164,165]
**PR-924**	β5i	An epoxyketone-based peptidyl selective inhibitor of β5i immunoproteasome, displays a much stronger inhibitory activity (β5c/β5i = 91) and blocks the growth of multiple myeloma in vitro and in vivo.	Multiple myeloma			[166,167]
**KZR-616**	β5i, β2i and β1i	The only epoxyketone-based peptidyl immunoproteasome selective inhibitor tested in clinic so far			Systemic lupus erythematosus (NCT03393013)	[168]

### 3.2. Ubiquitin-Conjugating Enzymes (E2s) Inhibitors

E2 enzymes, which act as intermediates between the E1 and E3 proteins, determine the type of the polyubiquitin chain linkage. However, each E2 needs to associate and cooperate with a specific set of E3s; the more applicable approach is to block the E2–E3 association through the inhibition of E3s. Thus, E2 enzymes have received far less attention as drug targets in discovering novel proteasome inhibitors. Among the few compounds developed, CC0651—an allosteric inhibitor of human E2 enzyme hCdc34—causes large-scale structural rearrangements that affect the discharge of ubiquitin acceptor lysine residues [169]. NSC697923 is another inhibitor targeting the Ubc13–Uev1A E2 enzyme and blocks the formation of the E2–Ub thioester conjugate, further inhibiting the activation of NF-κB signaling, leading to the reduced proliferation and viability of cancer cells [170]. Please see Table 1.

### 3.3. Ubiquitin Ligases (E3s) Inhibitors

So far, more than six hundred E3 enzymes have been discovered and found to diversely regulate the activity of downstream substrates [171]. E3 ligases are closely and specifically related to fundamental cellular processes in human cancers by regulating the degradation of tumor promoters or suppressors, thus inhibiting the activity of tumor-related E3 ligases could enhance the efficiency of cancer therapy by minimizing off-target side effects. More importantly, unlike E1 or E2, E3 ligases exhibit high specificity to a certain substrate. In this scenario, the targeting of E3 can be achieved in several ways, including through the inhibition of its expression levels, altering of its subcellular localization and via preventing its proper assembly [13,40] and/or interaction with cellular substrates [172,173]. The current main approach for the development of anti-E3-based therapies is via small-molecule screening technologies. As such, a number of studies have identified compounds targeting different E3 ligases and further impact the function and activity of UPS. 

The most studied E3 ligase is MDM2, which negatively regulates p53 and is important for cell survival [174,175,176], losing Mdm2 is reported to induce cell death both in vitro and in vivo in a p53-dependent manner [177]. Nutlin-3a, the first molecule described targeting MDM2, inhibits the interaction between Mdm2 and p53 [178], eventually arrests cell cycle, inhibits growth of cancer cells, and induces cell death in vitro and in vivo. The derivatives of nutlin-3a, such as (R05503781) [179] and RG7112 (R05045337) [180], have exhibited greater activities in vitro; however, the first result report of RG7112 in clinical trials for the treatment of liposarcoma is not so encouraging, due to the reason that even the expression levels of p53 and p21 increased in response to the treatment of RG7112, out of twenty patients, only one (1/20) showed a partial response. Furthermore, even though it specifically inhibits the activity of MDM2, RG7112 still shows relatively severe side effects including thromboycytopaenia and neutropaenia [180]. This illustrates one of the main concerns about the activation of p53 on normal cells when p53 is stabilized in therapies.

Another E3 ligase which has shown potential as a drug target is a protein family named inhibitors of apoptosis (IAPs). GDC-0152 and SM-406 are potent and orally bioavailable SMAC mimetic and an antagonist of the inhibitor of IAPs. It has good oral bioavailability and is highly effective in the induction of apoptosis in xenograft tumors and is capable of completely inhibiting tumor growth [181,182]. However, the clinical trial was terminated at phase I in 2009 without further notice.

Several other E3 ligases have also been considered as targets for the development of novel anticancer drugs (Please see Table 1). However, it is still worth noticing that: (a) E3 ligases can act as both tumor suppressors and promoters in a substrate-dependent and context-dependent manner due to the complex regulation of cellular activities; thus, targeting a specific E3 ligase requires a deep understanding its mechanism in both tissue-dependent and tumor-dependent conditions, (b) the ideal inhibitors would only disrupt the interactions of an E3 with substrates that are critical to cancer biology but not normal cell populations. Efforts have been made by targeting E3 ligases; however, the inhibitory specificity on cancer cells still needs to be stressed when considering the normal tissue, (c) since the mechanism underlying E3 ligases regulating cellular processes is complex, it is of paramount importance to understand how this post-translational modification mediated by E3 ligases is actively regulated, not only in cancer cells, but also in normal tissues. 

### 3.4. Inhibitors Targeting the Proteasome Complex

**Figure 3:**
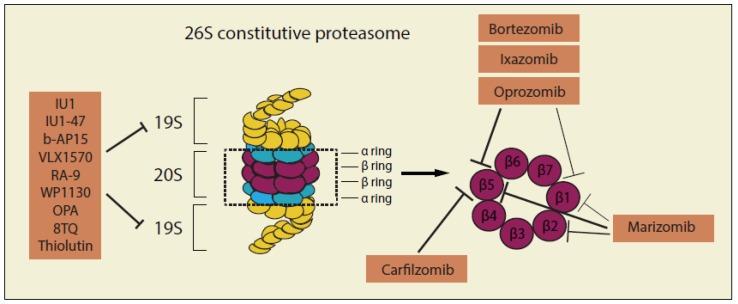
Structure and inhibitors of the 26S proteasome complex. The 26S complex consists of a 20S catalytic core particle which is capped at both ends by 19S regulatory particles. Inhibitors targeting the proteasome complex are generally divided into two groups: inhibitors of 20S catalytic core particle and inhibitors of 19S regulatory particles.

Proteins that have been adequately polyubiquitinated (Ub ≥ 4) are further identified and broken down by the 26S macromolecular proteasome complex. The 26S complex consists of a 20S catalytic core particle that is capped at both ends by 19S regulatory particles [47], thus inhibitors targeting the proteasome complex are generally divided by two groups: inhibitors of 20S catalytic core particle and inhibitors of 19S regulatory particles (Figure 3 and Table 2). 

### 3.5. Inhibitors of 20S Proteasome Catalytic core Particle 

#### 3.5.1. Bortezomib: First-in-Class Proteasome Inhibitor 

Bortezomib (BTZ, Velcade^®^) inhibits the chymotrypsin-like activity of the proteasome by reversible binding to the β5 subunit of the 20S proteasome thus impedes all proteasomal activity and leads to accumulation of polyubiquitinated proteins in cells [98,99]. Supported by strong preclinical data, BTZ entered an early phase clinical trial in late 2001. In early-phase clinical trials BTZ was generally well tolerated and showed mild adverse events, such as moderate fever and fatigue which were generally accompanied by thrombocytopenia and peripheral neuropathy [183]. Because of the promising results from early phase clinical trials, BTZ received US FDA fast-track approval for the treatment of relapsed and refractory MM in 2003 [184]. Later, it was approved in clinical trials for relapsed mantle cell lymphoma and diffuse large B-cell lymphoma [185,186]. When combined with other therapeutic anticancer agents, BTZ could achieve even better clinical efficacy, thus leading to a full US FDA approval in 2005 as a second-line MM therapy [187,188], and as a first-line therapy for patients with newly diagnosed MM after only three years [189].Unfortunately, the therapeutic window of BTZ is relatively narrow and toxic side effects gradually started to appear, ranging from peripheral neuropathy, myelosuppression and cardiotoxicity. This is probably due to the accumulation of misfolded proteins in normal tissues [190,191,192]. Additionally, there is a relatively high incidence of developing an acquired resistance during treatment with BTZ. This is mainly explained by the increased mRNA and protein expression of the β5-subunit of the proteasome that mutations occur in the subunit binding of BTZ, constitutive activation of the NF-κB signaling pathway and upregulation of the endoplasmic reticulum (ER) chaperone protein GRP78 and P-glycoprotein, as well as a multidrug resistance protein [193,194,195]. Thus, a second proteasome inhibitor, Carfilzomib, with the same target proteasome, has been developed and further approved by the FDA in 2012 for the treatment of multiple myeloma for patients who have shown a resistance to BTZ [196].

#### 3.5.2. Carfilzomib: Second-in-Class Proteasome Inhibitor

Carfilzomib (CFZ, PR-171, Kyprolis^®^) was initially discovered by the identification of the proteasome as the major target of the natural product epoxomicin [197]. Then a library of epoxomicin analogy was set up and a lead candidate YU-101 was identified due to its potent anticancer activities [198,199]. After a structure modulation, CFZ was further developed and displayed very solid preclinical results as a proteasome inhibitor [200]. Structurally, CFZ has a different structure (tetrapeptide epoxyketone) comparing with BTZ (dipeptide boronate) [201] and it forms an irreversible, covalent bond with proteasome catalytic subunits, predominantly β5. In 2005, phase I clinical trials with CFZ began and the drug was successfully investigated in additional clinical trials [105,106]. This included phase III clinical trials where it was shown that CFZ was effective in patients with relapsed and BTZ-chemoresistant disease [202,203]. Owing to its more selective mechanisms of action, CFZ had fewer side effects as compared to BZT including less pronounced neuropathy. Of note, CFZ showed some mild cardiotoxicity but these events were generally manageable and reversible [203,204]. The drawbacks of CFZ are that the drug is poorly soluble in water, not orally available and requires a large (50-fold) excess of cyclodextrin for injectable preparations. These problems, along with the onset of chemoresistance, warrants for developing additional next-generation proteasome inhibitors which could overcome drug resistance generated from a continuous treatment of BTZ or CFZ. 

#### 3.5.3. Ixazomib: First Oral Proteasome Inhibitor Drug

Both BTZ and CFZ can only be administered via subcutaneous or intravenous injection, thus there is a need to develop orally available proteasome inhibitors. In 2015, Ixazomib (IXZ, MLN9708, Ninlaro^®^) received its US FDA approval as the first orally bioavailable proteasome inhibitor drug [113,114]. IXZ orally administered once a week (4 mg on days 1, 8, and 15 of 28-day cycles) in combination with lenalidomide plus dexamethasome, has now been approved in 40 countries including the USA and the EU for the treatment of MM patients who have received either BTZ or CFZ in their previous treatments [205,206]. IXZ displays an encouraging positive safety profile including no effects on the mitochondrial serine protease HtrA2/Omi which was found to be an off-target of BTZ and the main reason for BTZ-related neurophaty [113,207,208]. Giving these promising results, the effect IXZ is currently under investigation as either a single or combined therapeutic approach for a number of cancers. The results of this trial will answer the question of whether IXZ has therapeutic advantages over BTZ or CFZ especially in patients affected by MM.

#### 3.5.4. Oprozomib: A Structural Homologue of CFZ

Oprozomib (OPZ, ONX-0912, PR-047), OPZ is a structural homologue of CFZ but more orally available. The drug is currently being investigated in several clinical trials in patients with hematological malignancies. The first promising results of these clinical trials show an overall response rate of 25% and 27.3% in patients with MM relapsed which had previously received BTZ- and CFZ-based therapy, respectively [120,121]. Of note, an Ib trial showed moderate to severe side-effects including vomiting, suggesting that the correct dosing of OPZ is crucial to avoid too high concentrations that are likely to result in proteasome inhibition in non-targeted tissues, especially tissues in the GI tract [209,210]. 

#### 3.5.5. Marizomib

Marizomib (NPI-0052, Salinosporamide A) is derived from the bacteria Salinospora tropica and is currently being investigated as a novel orally available proteasome inhibitor [211]. Unlike other peptide-based proteasome inhibitors, marizomib has a β-lactone-γ-lactam bicyclic ring structure without a linear peptide backbone [126,212,213]. Surprisingly, Marizomib could irreversibly inhibit the activity of proteasomes at the nanomolar range in MM cells [214,215]. Preclinical studies conducted with Marizomib show that following intravenous administration, proteasomal activity was inhibited in various tissues but slowly recovered over time with a course of recovery depending on the tissue but generally persisting up to 72 hours in blood [215,216]. Marizomib was shown to selectively affect the cell viability of MM and Lymphocytic Leukaemia (CLL) cancer cells and have less toxicity on normal cells as compared to BTZ [215,217]. Marizomib was also effective in killing MM cells derived from patients with resistance to BTZ [124,215,218]. Clinical trials in patients with refractory or relapsed MM showed an overall response rate of 11% when marizomib was used as monotherapy [128], with the rate increasing to 53% when the drug was combined with pomalidomide and low-dose dexamethasone [218]. Note worthily, marizomib treatment has been associated with some central neurotoxicity and has been shown to induce apoptosis glioma cells, these effects indicating that the drug penetrates the blood–brain barrier and that its use would be worth exploring as a potential treatment for brain cancer [124,219].

### 3.6. Inhibitors of 19S Proteasome Regulatory Particles

Acquired drug resistance, which is common in many cancer therapies, is also a major hurdle in proteasome inhibitor-based chemotherapies. MM patients who initially respond to proteasome inhibitors targeting 20S CP almost always eventually develop a resistance. There are currently few effective treatment options left, once patients relapse with MM refractory to proteasome inhibitor-based therapy. Current studies on the mechanism of resistance to the 20S CP proteasome inhibitors has provided important guidance for the screening of novel proteasome inhibitors that can potentially overcome the resistance generated by currently available proteasome inhibitors. Inhibitors of 19S proteasome regulatory particles (Table 2), especially the deubiquitinases (DUBs), are believed to be one of the potential targets for overcoming the acquired drug resistances of proteasome 20S inhibitors, as they have different target sites sites [131,220,221]. UCHL5 (or UCH37), USP14 and POU1 (Rpn11) are the three DUBs of the 19S proteasome that have been massively investigated and targeted due to their great potency on cancer cells [132,222,223,224,225].

#### 3.6.1. IU1 

IU1, a pyrrolyl pyrrolidinyl-ethanone, is the first USP14-specific inhibitor discovered from a high-throughput assay [226]. Its structure indicates that the drug targets the thiol group in the active site cysteine in USP14 proteases. Studies further suggested that the inhibition of USP14 decreases the proliferation of breast cancer cells [129]. IU1-47, an analog of IU1, was synthesized and tested in cultured neurons. It was reported that IU1-47 was tenfold more potent than the parental IU1. It has also been reported that IU1-47 causes a degradation of wild-type tau in neurons at a significantly higher rate than IU1 due to its extra targets on lysine-174 in tau protein, which may contribute to the higher specificity and efficacy [227]. A recent study has tested IU1-47 in lung cancer and proved that the inhibition of proteasome USP14 by IU1-47 could significantly decrease cell proliferation, migration, and invasion in lung cancer [130].

#### 3.6.2. b-AP15

b-AP15 was discovered as an inhibitor targeting both UCHL5 and USP14 in 19S proteasome regulatory particles. The α,β-unsaturated carbonyl group is thought to be directly involved in the Michael addition with the thiol in the active site cysteine [131,132]. Gene expression signatures of b-AP15 from the connectivity map database suggested that b-AP15 shared similarities with other potent proteasome inhibitors, such as BTZ. However, b-AP15 and BTZ target different subunits of proteasome, and due to the different inhibition of the proteasome, b-AP15 is able to disrupt the protect mechanism of forming aggresomes in cancer cells exposed to BTZ [228]. Additionally, the data showed that b-AP15 induced a dose-dependent aggregation of conjugated ubiquitin, suggesting inhibition of the degradation activity of the DUBs [131]. Further studies indicated that b-AP15 is an inhibitor of both USP14 and UCHL5 and has an IC50 value of 2.1 μM when using purified 19S proteasome [131]. It has been shown that b-AP15 could overcome BTZ induced resistance in MM cell lines by activating caspase to further induce apoptosis relating to an upregulation of oxidative stress [229]. In vivo studies revealed that tumor growth was blocked by b-AP15 in several human xenografts [132]. 

#### 3.6.3. VLX1570

VLX1570 was developed as an analog of b-AP15 to increase the in vivo selectivity and efficacy [143]. Structurally, the α,β-unsaturated carbonyls or the Michael acceptor was not modified from b-AP15. However, the structure of VLX1570 differs in that two 4-nitrobenzylidne groups in b-AP15 were replaced by two 4-fluoro-3-nitrobenzylidene groups in VLX1570, thus the electron-withdrawing property on the side aryls is enhanced. Results suggested an increased inhibition of USP14 by VLX1570 compared with b-AP15. Adversely, the competitive binding assay using Ub-VS showed that the analog displayed a greater specificity to USP14 rather than UCHL5 compared with b-AP15 [143]. In vivo studies on MM cells revealed that VLX1570 was more effective than b-AP15 in inhibiting tumor progression in mice [142]. As there is a strong outcome of VLX1570 in xenograft models, VLX1570 was then promoted to clinical trials.

#### 3.6.4. RA-9

RA-9 is another compound with a structure very similar to b-AP15. RA-9 belongs to the family of chalcone-based derivatives with α,β-unsaturated carbonyls that are thought to react with the sulfurs in the active site cysteine [230,231,232]. RA-9 was shown to have inhibitory properties for proteasome-associated DUBs. There was a dose-dependent relationship found in a Ub-AMC assay of 19S DUBs treated with RA-9, which supports the proposed specificity by the authors [233]. Moreover, RA-9 was reported to selectively induce apoptosis in primary cultures from donors. Loss of cell viability following RA-9 exposure was associated with an unfolded protein response in ovarian cancers. In vivo treatment with RA-9 retards tumor growth, increases overall survival, and was well tolerated by the host [233].

#### 3.6.5. WP1130 

WP1130 is described as a small molecule activating a novel Bcr/Abl destruction pathway further inducing the apoptosis of chronic myelogenous leukemia, which leads to aggresome formation. It was found that WP1130 can directly inhibit USP9X as well as DUBs of UCHL5, and USP14. As USP9X inhibition has been linked to apoptosis and prevention of drug resistance in malignancies through Mcl-1 degradation, WP1130 is thought to target a Bcr-Abl-/Mcl-1-specific pathway as a USP inhibitor, suggesting a capacity for cancer treatment [148,149]. The inhibition of deubiquitinases by the compound WP1130 has further been reported to inhibit ULK1 activity and block the autophagic flux [234].

#### 3.6.6. RA190

RA190 is an orally available bis-benzylidine piperidone derivative that inhibits the proteasome functions by covalently binding to cysteine 88 of ubiquitin receptor RPN13 in the 19S regulatory particle [235]. Biophysical analyses in combination with cell-based assays indicate that RA190 directly binds and inactivates Uch37 [236]. This compound can trigger the rapid accumulation of polyubiquitinated proteins followed by proteotoxic stress and apoptosis in cancer cells [235]. RA190 was originally described to be effective even in MM cells resistant to BTZ and has been preclinically tested in several cancer models including MM, ovarian, cervical and gastric cancers either alone or in combination with other chemotherapy agents [237,238,239,240,241].

#### 3.6.7. Ortho-Phenanthroline (OPA)

1,10-Phenanthroline, also known as OPA, is a zinc ion chelator [155]. While USPs and UCHs mostly do not contain an incorporated metal, Ubiquitin carboxyl-terminal hydrolase RPN11 does have a zinc-bound active site. It has been reported that OPA inhibits the activity of purified RPN11 [156]. Furthermore, it was shown that OPA does not affect proteasome activity when added to proteasome harboring RPN11-mutated extract compared with unmodified extract, which further supports that OPA specifically targets RPN11 [156]. Research on the efficacy of OPA as a potential cancer treatment has been started in MM. Studies indicate that OPA’s metallopeptidase inhibition activity is linked to apoptosis in myeloma cell lines including cell lines, which were BTZ resistant [157].

#### 3.6.8. Quinoline-8-Thiol/Capzimin

Quinoline-8-thiol (8TQ) is a first-in-class inhibitor with a strong inhibition specificity to RPN11 of 19S proteasome subunit. A fragment-based drug discovery approach was instrumental in the identification of the RPN11 inhibitor. Studies describe that slight modifications to 8TQ may increase its inhibition activity. 8TQ and its analogs are proposed to chelate the zinc ion bound to the active site of RPN11. 8TQ has been proposed as a possible novel treatment for MM and other cancers. 8TQ and its associated compounds were shown to be potent apoptosis inducers in MM cells. After the development of analogs of 8TQ, a derivative named ‘capzimin’ was selected for further investigation. Capzimin was shown to have more than fivefold selectivity for the metalloprotein RPN11. Capzimin stabilized proteasome substrates, induced an unfolded protein response, and reduced the proliferation rate of cancer cells, including those resistant to bortezomib. Proteomic analysis revealed that capzimin stabilized a subset of polyubiquitinated substrates. The identification of capzimin offers an alternative path to develop proteasome inhibitors for cancer therapy [162].

#### 3.6.9. Thiolutin

Thiolutin (THL) was originally discovered as an antibiotic that is able to inhibit bacterial and fungal RNA polymerases [242,243,244]. The latest data have indicated that the reduced form of THL is an inhibitor of JAB1/MPN/Mov34 (JAMM) domain-containing metalloprotease RPN11 by chelating Zn2+-ions, which are specifically toxic to cancer cells by hampering protein turnover and inducing ubiquitylation [163]. As with 8TQ, a reduced form of thiolutin harbors a totally different chemical structure and targets distinct components of the UPS which merits further investigation of the mechanism underlying and provides up-and-coming orientations of overcoming the obstacle of drug resistance to BTZ in cancer therapies. 

## 4. Targeting the Ubiquitin–Proteasome System (UPS) and Immune System in Cancer Therapies

**Figure 4:**
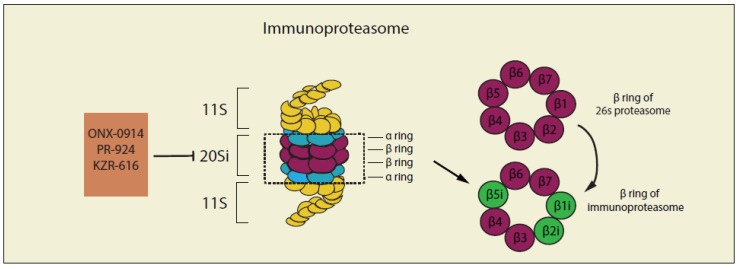
Structure and inhibitors of the immunoproteasome complex. In immunoproteasome, β5 (PSMB5), β1 (PSMB6), and β2 (PSMB7) of the constitutive proteasome complex are replaced by their respective inducible counterparts β5i (LMP7) β1i (LMP2), and β2i (MECL-1), under inflammatory conditions and certain pathological states, including cancer. ONX-0914, PR-924 and KZR-616 are reported as selective inhibitors of immunoproteasome (Table 3).

Cells in the immune system express an inducible form of the proteasome called the immunoproteasome [245] which has different compositions of proteasome 20S core compared with the constitutive proteasome complex described above (Figure 4). In immunoproteasome, β5 (PSMB5), β1 (PSMB6), and β2 (PSMB7) of the constitutive proteasome complex are replaced by their respective inducible counterparts β5i (LMP7) β1i (LMP2), and β2i (MECL-1), under inflammatory conditions and certain pathological states including cancer [246]. It has been reported that immunoproteasomes are more efficient than constitutive particles in degrading polyubiquitinated proteins and are essential for removing damaged proteins in inflammatory states because they can efficiently digest misfolded proteins that form aggresome-like protein conjugates [247]. Several studies [248,249,250] have revealed that the expression level of immunoproteasome is much higher compared with that of constitutive subunits in B-cell malignancies, indicating the importance of the immunoproteasome in the regulation of protein homeostasis of hematologic diseases [251] and suggesting that targeting the function of immunoproteasomes could be a possible strategy for the treatment of cancer. 

Another reason for targeting the immunoproteasomes in the cancer setting is because of its essential role in acquired resistance to bortezomib. The clinical impact of acquired resistance has been demonstrated in poor responses of MM patients who were re-treated with bortezomib [252]. To understand the underlying possible mechanisms of bortezomib resistance, in vitro cell line models of hematologic malignancies have been developed in which acquired resistance to bortezomib was developed by chronic exposure to gradually increasing bortezomib concentrations [193,253]. These bortezomib-resistant cell lines displayed a cross-resistance to other proteasome inhibitors that target constitutive proteasome subunit β5 (PSMB5) of the proteasome. Furthermore, these bortezomib-resistant cell lines were characterized by an increased expression of the constitutive proteasome subunit β5 (PSMB5) harboring mutations in the bortezomib-binding pocket, along with a decreased expression of non-mutated immunoproteasome subunits [193]. Original studies showed that inflammatory cytokines such as IFN-γ and TNFα were efficient inducers of immunoproteasomes in MM cell lines [254]. Functional studies indicated that exposure to interferon-γ (IFN-γ) enhanced bortezomib-sensitivity in B-cell lines by 50% [255]. From a therapeutic perspective, this could indicate that modulating the expression balance of immunoproteasomes and constitutive proteasomes could re-confer the sensitivity of cancer cells to bortezomib or develop the next generation of immunoproteasomes inhibitors, a strategy for the treatment of cancer (Table 3). 

### 4.1. Non-Selective Inhibitors of Immunoproteasome

Most inhibitors of the constitutive proteasomes, such as the 20S CP inhibitors Bortezomib, Carfilzomib and Ixazomib are non-selective immunoproteasome inhibitors [256]. For example, the inhibitory activity of Bortezomib against proteasome β5c is IC50 7nM and against immunoproteasome β5i is IC50 3.3nM [257]. Unfortunately, resistance to 20S CP inhibitors is common and is characterized by an upregulated expression of the constitutive proteasome subunit β5 (PSMB5). Therefore, more specific immunoproteasome inhibitors are needed to provide therapeutic opportunities when resistance to proteasome inhibitors occurs.

### 4.2. Selective Inhibitors of Immunoproteasome 

#### 4.2.1. ONX-0914

ONX-0914 (also called PR957) is the first epoxyketone-based peptidyl immunoproteasome-selective inhibitor. ONX-0914 displayed a higher inhibitory activity towards immunoproteasome β5i (IC50 5.7 nM) as compared to the constitutive proteasome β5c subunit (IC50 54 nM) [258]. ONX-0914 has been proven to be effective in the treatment of inflammatory disorders by specifically targeting immunoproteasome [164,259]. This indicates a therapeutic potential for anticancer therapies where the activity of immunoproteasome is upregulated. 

#### 4.2.2. PR-924

PR-924 is another tripeptide epoxyketone immunoproteasome β5i-selective inhibitor. As compared to ONX-0914, PR-924 displayed a much stronger inhibitory activity towards immunoproteasome (IC50 2.5 nM) compared to the constitutive proteasome β5c subunit (IC50 227 nM) [256]. PR-924 was shown to inhibit the growth of multiple myeloma cells in vitro and in vivo with no significant side effects on normal peripheral blood mononuclear cells [167]. A further study also demonstrated that PR-924 is effective in killing bortezomib-resistant leukemia cells [166]. This indicates a therapeutic opportunity when bortezomib-resistance occurs. 

#### 4.2.3. KZR-616

KZR-616 is the third tripeptide epoxyketone immunoproteasome-selective inhibitor developed based on the optimization of the inhibitors ONX-0914 and PR-924. KZR-616 is currently the only immunoproteasome-selective inhibitor that was approved by the FDA and tested in clinic [168]. It is worth noting that the derivatives of KZR-616 also display an improved inhibitory activity towards the β1i subunit of immunoproteasome, with an IC50 0.425 nM towards β1i, but an IC50 > 250 nM towards β1c (β5c/β5i > 602) [256].

## 5. Concluding Remarks

The Ubiquitin–Proteasome System (UPS) plays an important role in cancer initiation and progression as well as in the onset of chemoresistance. This makes the UPS an attractive, albeit complex, molecular target for cancer treatment. A number of small-molecule inhibitors of various components of the UPS have been successfully used in a clinical setting as anti-cancer agents, including as a first line of treatment. However, a number of challenges remain when developing cancer drugs targeting the UPS. These include (a) drug resistance acquired after the continuous treatment of proteasome inhibitors, (b) limited efficacy in the treatment of solid tumors, and (c) the yet to be determined clinical efficacy of immunoproteasome inhibitors for the treatment of human cancers. Despite these challenges, the current literature indicates that the development of anticancer drugs that target single or multiple components of the UPS for cancer treatment is worth further exploration.

## Figures and Tables

**Table 1:** Inhibitors targeting E1s, E2s and E3s.

Compounds	Target	Modes of Action	Targeted Cancer Types in Preclinical Studies	Targeted Cancer Types in Clinical Studies or Therapies	Other Disease	Ref.
**Inhibitors targeting E1s of the UPS**						
**PYR-41**	UBA1	Irreversibly binds to the active cysteine in UBA1 and kill tumor cells by inhibiting cytokine-induced NF-κB activation, and promoting p53 accumulation	Prostate cancer Thyroid cancer		Hypertensive heart diseases/ Sepsis	[1,2,3,4,5,6]
**MLN4924**	NAE	Covalently binds the nucleotide-binding site of NAE and generates a NEDD8-MLN4924 adduct that further undermines protein turnover leading to apoptosis in cancer cells	Liver cancer Pancreatic cancer	Acute Myelogenous Leukemia (AML) Multiple Myeloma Lymphoma Melanoma Lung Cancer Mesothelioma	Pulmonary inflammation/ Ipopolysaccharide-induced kidney damage/ Spinal cord ischemia-reperfusion injury/ Myelodysplastic Syndromes	[7,8,9,10,11,12,13,14,15,16]
**Inhibitors targeting E2s of the UPS**						
**CC0651**	hCdc34	An allosteric inhibitor of human E2 enzyme hCdc34, causes large-scale structural rearrangements and affects the discharge of ubiquitin to acceptor lysine residues	Prostate cancer Colon cancer		No reported applications	[17]
**NSC697923**	Ubc13–Uev1A E2	Blocks the formation of the E2–Ub thioester conjugate and inhibits the activation of NF-κB signaling leading to reduced proliferation and cell viability	Melanoma B-cell lymphoma Neuroblastoma Colorectal Cancer		Diabetic nephropathy	[18,19,20,21,22]
**Inhibitors targeting E3s of the UPS**						
**Nutlin-3a**	Mdm2	Competitively binds the Mdm2-P53 interacting site, activates P53 pathway, and thus results in cell cycle arrest, cell death, and growth inhibition	Acute/Chronic lymphocytic leukemia Hodgkin lymphoma Pancreatic cancer Glioblastoma Sarcoma Colon cancer Breast cancer Ovarian cancer Lung cancer Ewing sarcoma		Pulmonary arterial hypertension	[23,24,25,26,27,28,29,30,31,32,33,34,35]
**RG7388 (R05503781)** **RG7112 (R05045337)**	Mdm2	The derivatives of nutlin-3a and Inhibits Mdm2-P53 binding site		Acute myeloid leukemia Relapsed or refractory Acute myeloid leukemia Multiple myeloma Relapsed multiple myeloma Glioblastoma Ovarian cancer Childhood sarcoma Neuroblastoma Breast cancer Lung cancer	Polycythemia vera/ Essential Thrombocythemia	[36,37,38,39,40,41,42,43]
**GDC-0152** **SM-406**	IAPs	Potent and orally bioavailable SMAC mimetic and antagonists of the inhibitor of IAPs with highly effective in induction of apoptosis in xenograft tumors, and is capable of inhibition of tumor growth	Osteosarcoma Leukemia Thyroid cancer Glioblastomas Breast cancer		No reported applications	[44,45,46,47,48]
**SCF-12**	FBW7	Blocks the substrate-binding pocket and impedes substrate recognition via inhibiting Cdc4 thus hinders tumor progression in colon and prostate cancers	Colon cancer Prostate cancer		No reported applications	[49]
**Oridonin**	FBW7	Targets FBW7-c-Myc pathway and activates GSK-3, decreases c-Myc and induces apoptosis in leukemia and lymphoma cells	Myelogenous leukemia Breast cancer		Myocardial ischemia Reperfusion injury	[50,51,52]
**Compound #25**	SKP2	Directly binds SKP2, selectively suppresses Skp2 E3 ligase activity and exhibits potent antitumor activities in multiple animal models	Prostate cancer		No reported applications	[53]
**NAHA**	Cdc20	Decreases Cdc20 expression and inhibits tumor proliferation in vitro and in vivo associated with the induction of apoptosis	Breast cancer		No reported applications	[54]
**CM_11_-1**	E6AP	Acts as an E6AP inhibitor that prevents polyubiquitination of Prx1 and p53 in E6-independent and E6-dependent manner	Only in RaPID System cell free system		No reported applications	[55]
